# Toward identifying specific roles for G-protein β and γ subunit variants in olfactory reception

**DOI:** 10.3389/fncel.2013.00114

**Published:** 2013-07-17

**Authors:** Tamara Boto, Esther Alcorta

**Affiliations:** ^1^Departamento de Biologia Funcional (Genetica), Facultad de Medicina, Universidad de OviedoOviedo, Spain; ^2^Department of Neuroscience, The Scripps Research InstituteJupiter, FL, USA

G-proteins mediate many cellular signaling processes; some are restricted to certain tissues or cell types, whereas others are involved in more general activities. For example, information regarding a change in the concentration of peptides, hormones, lipids, neurotransmitters, ions, odorants and tastants or an influx of photons to the eye can be transmitted to a cell via G-proteins.

Heterotrimeric G proteins are composed of three subunits: Gα, Gβ and Gγ. When activated, the Gα subunit binding GTP, and the Gβγ heterodimer act on their effectors. In both vertebrates and invertebrates, many genes encode different variants of each subunit. In mammals, 20 genes encode the Gα, 5 Gβ, and 12 Gγ subunits (Malbon, 2005; Dupre et al., 2009), and there is also a considerable amount of variability in more simple organisms, such as *Drosophila melanogaster*, with 6 genes for Gα, 3 for Gβ and 2 for Gγ (Wolfgang et al., 1990; Yarfitz et al., 1991; Schulz et al., 1999; Boto et al., 2010).

Expression studies generally offer basic information on the possible biological function of gene products and have been extensively applied to G-proteins in many species. Previous reports have confirmed that gene expression is cell specific in some cases. For example, in *Drosophila*, the Gαq-1 isoform (Lee et al., 1994) and the Gβ76C subunit (Yarfitz et al., 1991) were found to be specifically expressed in photoreceptor cells, highlighting their role in phototransduction.

The possibility of relating specific expression of certain genes (specially for the less known Gβ and Gγ subunits) with particular functions in a comprehensive way is both a very interesting and hot issue for very different fields (see for example, O'Neill et al., 2012; El-Haibi et al., 2013). The attempt to relate particular Gβ and Gγ variants with olfactory reception tissues in mice is in the basis of the article by Sathyanesan et al. recently published in *Frontiers in Cellular Neuroscience* (2013, 7, 84).

In many vertebrates, olfactory reception is mediated by odorant receptors that belong to the G-protein-coupled receptor (GPCR) family (Mombaerts, 1999).

The expression pattern and functional roles for Gα proteins in olfactory reception have been deeply studied. Golf was found to be highly and almost exclusively expressed in olfactory receptor neurons (ORNs) of the main olfactory epithelium (MOE) (Jones and Reed, 1987), and a lack of Go in the vomeronasal organ (VNO) of mice elicits behavioral deficits (Chamero et al., 2011). However, the Gβγ subunits are not well-studied in mice, and only a few reports refer to the gene or protein expression of particular variants (Kulaga et al., 2004; Lin et al., 2007; Kerr et al., 2008; Li et al., 2013).

The Gβγ heterodimer is a functional structure that, unlike Gα, does not change its conformational state when it dissociates from the heterotrimer. *In vitro* studies show a high variability of possible Gβ and Gγ combinations, though the possibilities are more restricted in the native situation (Milligan and Kostenis, 2006).

In their paper in *Frontiers in Cellular Neuroscience*, Sathyanesan et al. (2013) performed a comprehensive study of the expression pattern of all Gβ- and Gγ-encoding genes (17) in mice olfactory receptor epithelia, MOE and VNO, in adult animals and also at different postnatal stages.

To this end, the researchers analyzed gene expression by RT-PCR and quantitative PCR using RNA extracted from both organs and designing specific primers for each Gβ and Gγ subunit based on the 3′ UTR region in an attempt to overcome possible homology. The authors reported strong expression of the β1, γ8, and γ13 genes in MOE, confirming previous results from other studies (Lin et al., 2007; Kerr et al., 2008), and also detected for the first time the expression of Gβ2,4 and 5 and Gγ2,3,5,10,11, and 12 in this tissue. A quantitative analysis confirmed that β1, γ8 and γ13 are the most abundant transcripts in the main olfactory epithelium of the mouse. Sathyanesan et al. similarly analyzed the expression of the Gβ and Gγ subunits in VNO, and their results showed the expression of only Gβ1 among the Gβ group (and perhaps a very weak signal for Gβ2), and the strong presence of Gγ2,3,8, and 13.

These data are based on the total RNA present in the organs. Thus, for further detail on the presence of distinct G proteins in different cell types in these olfactory organs, the authors performed *in situ* RNA hybridization experiments (RISH) to localize the Gβ and Gγ transcripts to specific cells.

Although the RISH results did not consistently agree with the data from PCR experiments, as explained in the manuscript, such a situation can be due to the different sensitivities of the techniques or to the inherent technical difficulties of each. Nevertheless, the RISH data are reliable, considering that they show the most restrictive results. Gβ1 appears to be the only variant expressed in MOE and VNO olfactory receptor neurons. With regard to Gγ subunits, some expression specificity was detected, as Gγ2 and Gγ12 were only localized to supporting cells. The authors performed double-labeled experiments to show that, in a considerable proportion of neurons in MOE, Golf and Gγ13 are expressed in the same cells, as are Gβ1 and Gγ13. Therefore, Sathyanesan et al. propose that these three subunits may be part of the same heterotrimer.

The authors also show convincing results regarding specific expression, depending on the cell type, of the Gγ subunits in VNO. VNO sensory neurons differentially express two types of Gα proteins: Gi2 in the apical layer and Go in the basal layer (Jia and Halpern, 1996). Sathyanesan et al. found 4 types of Gγ in VNO: Gγ2,3,8, and 13. Although all are expressed in the Gi2 layer, only was found to Gγ8 localize to the basal Go layer of neurons.

Further experiments testing the cellular location and protein interactions will be necessary to confirm these data, but the finding of gene expression specificity for some Gβ and Gγ subunits is an important step toward unraveling olfactory transduction in mammals and the role of G-proteins in the development of olfactory reception tissues.
